# Overexpression of LncRNA SNHG14 as a biomarker of clinicopathological and prognosis value in human cancers: A meta-analysis and bioinformatics analysis

**DOI:** 10.3389/fgene.2022.945919

**Published:** 2022-10-06

**Authors:** Bin Liu, Tingting Lu, Yongfeng Wang, Guangming Zhang, Liangyin Fu, Miao Yu, Kehu Yang, Hui Cai

**Affiliations:** ^1^ The First Clinical Medical School, Lanzhou University, Lanzhou, Gansu, China; ^2^ Key Laboratory of Molecular Diagnostics and Precision Medicine for Surgical Oncology in Gansu Province, Gansu Provincial Hospital, Lanzhou, Gansu Province, China; ^3^ Institution of Clinical Research and Evidence Based Medicine, Gansu Provincial Hospital, Lanzhou, Gansu Province, China; ^4^ The First Clinical Medical College of Gansu University of Chinese Medicine, Lanzhou, Gansu Province, China; ^5^ Evidence-Based Medicine Center, School of Basic Medical Sciences, Lanzhou University, Lanzhou, Gansu Province, China

**Keywords:** lncRNA, SNGH14, cancer, meta-analysis, prognostic lncRNA: long noncoding RNA, ceRNA: competing endogenous RNAs, SNHG14: small nucleolar

## Abstract

**Background:** SNGH14 is a newly discovered long non-coding RNA (lncRNA) highly associated with tumorigenesis. However, whether the level of SNHG14 is related to the prognosis of patients with different cancer types is unclear.

**Methods:** PubMed, Web of Science, Cochrane Library, and Embase were searched to identify eligible studies from inception to November 2021. The odds ratio (OR) and 95% confidence interval (CI) were utilized to analyze dichotomous variables, while the hazard ratio (HR) and 95% CI were used for survival outcomes. We also included trial sequential analysis (TSA) to assess whether the current evidence was sufficiently conclusive. Stata 15.0 and TSA 0.9 software were used for data analyses.

**Results:** A total of 21 studies involving 1,080 patients, mainly from China, were included. Our results revealed that high SNHG14 expression was associated significantly with poor overall survival (OS) [HR = 1.39; 95% CI: (1.06–1.83); *p* = 0.017]. In addition, elevated SNHG14 expression was related to tumor size (> 3.5 cm) [OR = 1.60; 95% CI: (1.20–2.14); *p* = 0.001], TNM staging [OR = 0.54; 95% CI: (0.40–0.71); *p* < 0.001], lymph node metastasis [OR = 1.86; 95% CI: (1.35–2.55); *p* < 0.001], differentiation grade [OR = 1.95; 95% CI: (1.36–2.80); *p* < 0.001], and distant metastasis [OR = 2.44; 95% CI: (1.30–4.58); *p* = 0.005]. However, no significant difference was observed between age [OR = 0.98; 95% CI: (0.72–1.35); *p* = 0.915] and gender [OR = 0.98; 95% CI: (0.72–1.35); *p* = 0.915] from the enhanced expression of SNHG14.

**Conclusion:** The current study revealed that overexpression of SNGH14 is associated with low OS rate and clinicopathological characteristics. SNGH14 can be a novel tumor marker that aids in tumor diagnosis, thereby improving patient prognosis.

## 1 Introduction

Cancer is one of the leading causes of global human death and a major public health problem (Lee and Sanoff, 2020). Cancer research has progressed significantly in recent years, but clinical outcomes are still not optimistic. The main reason is that cancers have not been effectively diagnosed and treated early, causing unsatisfactory clinical curative effects, which significantly impact the prognosis of patients (Hiatt and Beyeler, 2020). Therefore, there is an urgent need to discover novel biomarkers that can facilitate early diagnosis and prognostic evaluation among tumor patients (Wang et al., 2020).

Due to the development of whole-genome and transcriptome sequencing technology and the ENCODE project (Harrow et al., 2012), researchers have observed that most genome DNA is present in processed translation scripts. However, these translation scripts may not be translated into functional proteins, i.e., non-coding ribonucleic acid (ncRNA) (2012; Xie et al., 2019). Long non-coding ribonucleic acid (lncRNA) is a non-coding ribonucleic acid series with more than 200 nucleotides (Tang and Yang, 2020). Although lncRNA has almost no protein-coding ability, they have a vital role in regulating gene expression at various points during the transcription/translation process (Dong et al., 2018; Wanowska et al., 2018; Cheng et al., 2020). They have been considered promising markers for cancer prognosis, diagnosis, and development. The association between lncRNA and the carcinogenesis of many cancer types has been well established (Yu et al., 2019; Liu et al., 2020).

Small nucleolar RNA host gene 14 (SNHG14) is located on the human chromosome 15q11.2 and has been reported to accelerate tumor development in many malignant tumors (Hou and Mao, 2020). It is a proven proto-oncogene in various cancers, including lung and cervical cancer. It is essential in activating inflammatory microglia, sepsis-induced acute kidney injury, and LPS-induced acute kidney injury (Zhong et al., 2019; Jiang et al., 2021; Shi et al., 2021; Yang et al., 2021). Xie et al. (2020) identified that SNHG14 regulates E-cadherin expression by interacting with EZH2, enhancing the progression of pancreatic ductal adenocarcinoma. Recently, Liu et al. (2017) described that SNHG14 could be used as ceRNA to promote the initiation and clearance progress. It can overlap with the entire UBE3A gene and promoter, inhibiting UBE3A expression and causing neurogenetic diseases like Angelman syndrome (Sadikovic et al., 2014). However, the regulatory mechanism of SNHG14 remains unclear.

There is no prominent article to confirm the relationship between SNHG14 and cancer prognosis. In addition, several studies on SNHG14 have only obtained independent results due to limitations, including short follow-up durations. To provide better clinical guidance to clinicians, we have performed a meta-analysis of the existing literature to investigate the relationship between SNHG14 and clinicopathological features and patient prognosis in the present study.

## 2 Materials and methods

### 2.1 Registration

The present meta-analysis was conducted and reported based on the Preferred Reporting Items for Systematic Reviews and Meta-Analysis (PRISMA) guidance (Liberati et al., 2009). This study has also been registered in PROSPERO (No. CRD42021287397).

### 2.2 Search strategy

PubMed, Web of Science, Cochrane Library, and Embase were searched from inception to November 2021. The main combinations of search terms incorporated were as follows: (“small nucleolar RNA host gene 14” OR “SNHG14” OR “115HG” OR “IC-SNURF-SNRPN” OR “LNCAT” OR “NCRNA00214” OR “U-UBE3A-ATS” OR “UBE3A-AS” OR “UBE3A-AS1” OR “UBE3A-ATS” OR “UBE3AATS”) AND (“cancer” OR “carcinoma” OR “neoplasm” OR “tumor”) without any restrictions on population, and the reference list of included studies was also checked. The detailed search strategy is presented in Supplementary Material S1.

### 2.3 Incusion and exclusion criteria

The inclusion criteria were as follows: (a) assessment of the expression level of SNHG14 among cancer patients; (b) patients were divided into high and low expression groups; (c) outcomes describing overall survival (OS) and related clinicopathological parameters (including age, gender, tumor size, TNM staging, lymph node metastasis, differentiation grade, and distant metastasis) should be considered; (d) case-control studies (CCSs) or cohort studies (CS).

The exclusion criteria were: (a) reviews, case reports, conference abstracts, animal studies, fundamental experimental research, etc.; (b) duplicate publications; (c) studies lacking survival or clinicopathological data; (d) non-English language literature.

### 2.4 Data extraction

Two reviewers independently screened the literature and extracted data. Any disagreements were resolved by consulting a third investigator. The following information was extracted: first author, year of publication, country/region, cancer type, sample type, number of samples, detection method, cut-off values, outcomes, and follow-up periods.

The expression of LncRNA is detected by qRT-PCR, first using Trizol reagent to extract total RNA from tissues and cells. RNA integrity was evaluated by standard agarose/ethidium bromide gel electrophoresis. Then, total RNA was reverse transcribed into cDNA through a reverse transcription kit. The expression level of lncRNA was detected using fluorescence quantification. Glyeraldehyde-3-phosphate dehydrogenase (GAPDH) was used as an endogenous control. The results of expression level of lncRNA were analyzed using the comparative 2^–ΔΔCT^ method. The cut-off value generally chooses the mean or median (Huang et al., 2021).

### 2.5 Quality assessment and trial sequential analysis

Newcastle-Ottawa Scale (NOS) was utilized to assess the quality of included studies (Stang, 2010). The scale consisted of the following domains: selection of study groups, comparability, exposure (case-control study), or outcome (cohort study). The total NOS score ranged from 0 to 9, and the score of high-quality studies was ≥ 7. Any disagreements were resolved through consultation with a third investigator.

We used trial sequential analysis (TSA) to assess whether the current evidence was sufficient and sufficiently conclusive to prevent the risk of false-positive (type I) errors. In this review, the required information size was estimated through *α* = 0.05 (two sides), *β* = 0.20 (power 80%). The O'Brien-Fleming function was implemented. If the cumulative Z-curve crossed the monitoring boundary, a sufficient level of evidence for the effect of the intervention could have been reached. More research is required if the cumulative Z-curve crosses neither the traditional boundary nor the null region.

### 2.6 Data synthesis and statistical analysis

The dichotomous outcomes were represented as odds ratio (OR) and 95% confidence interval (CI). Engauge Digitizer V.4.1 software extracted HR and 95% CI from Kaplan-Meier (KM) curves. We determined logHR and SE logHR= [log(Upper Limit)-log (Lower Limit)]/3.92 and performed a meta-analysis through the inverse variance method to summarize OS (Parmar et al., 1998). Heterogeneity was assessed through Chi-square (χ2) test and I-square (*I* ^2^). A random-effects model was incorporated for data with significant heterogeneity (*P*_Q_ < 0.1 and *I* ^*2*^ > 50%). Otherwise, a fixed-effects model was utilized. Subgroup analysis was performed according to follow-up period, sample size, analysis method, and cancer type. Publication bias was assessed through Egger’s test and Begg’s funnel plot, and sensitivity analysis was conducted to identify the source of heterogeneity. Stata version 15.0 software (Stata Corporation, College Station, TX, United States) and TSA 0.9 (http://www.ctu.dk/tsa) software were used for data analyses, and *p* < 0.05 was considered statistically significant.

### 2.7 Target gene prediction and signal pathway network construction

The related genes for SNHG14 were retrieved from the MEM-Multi Experiment Matrix database (https://biit.cs.ut.ee/mem/index.cgi). Then, Gene Ontology (GO) and the Kyoto Encyclopedia of Genes and Genomes (KEGG) pathway enrichment analysis were conducted depending on SNHG14-related genes using R software (*p* < 0.05). Moreover, a signaling pathway network was constructed through the Cytoscape software.

### 2.8 The relationship between the expression of SNHG14 and tumor mutational burden or microsatellite instability in cancer

Using Spearman’s correlation analysis, we analyzed the association of tumor mutational burden (TMB) and microsatellite instability (MSI) with SNHG14 expression. The data were obtained from the TCGA database through the Genomic Data Commons (GDC) data portal website (https://portal.gdc.cancer.gov/) and statistically analyzed through R software v4.0.3.

### 2.9 Correlation of SNHG14 expression in cancer with immune cell infiltration

Newman and colleagues created CIBERSORT, which quantifies immune cell infiltration in all malignancies by estimating the number of specific cell types in mixed cell populations through gene expression data. Thus, we used CIBERSORT to evaluate the relationship between SNHG14 expression and immune cell infiltration with R packages “ggplot2,” “ggpubr,” and “ggExtra.” (*p* < 0.001 was the cut-off value).

## 3 Results

### 3.1 Study selection

**FIGURE 1:**
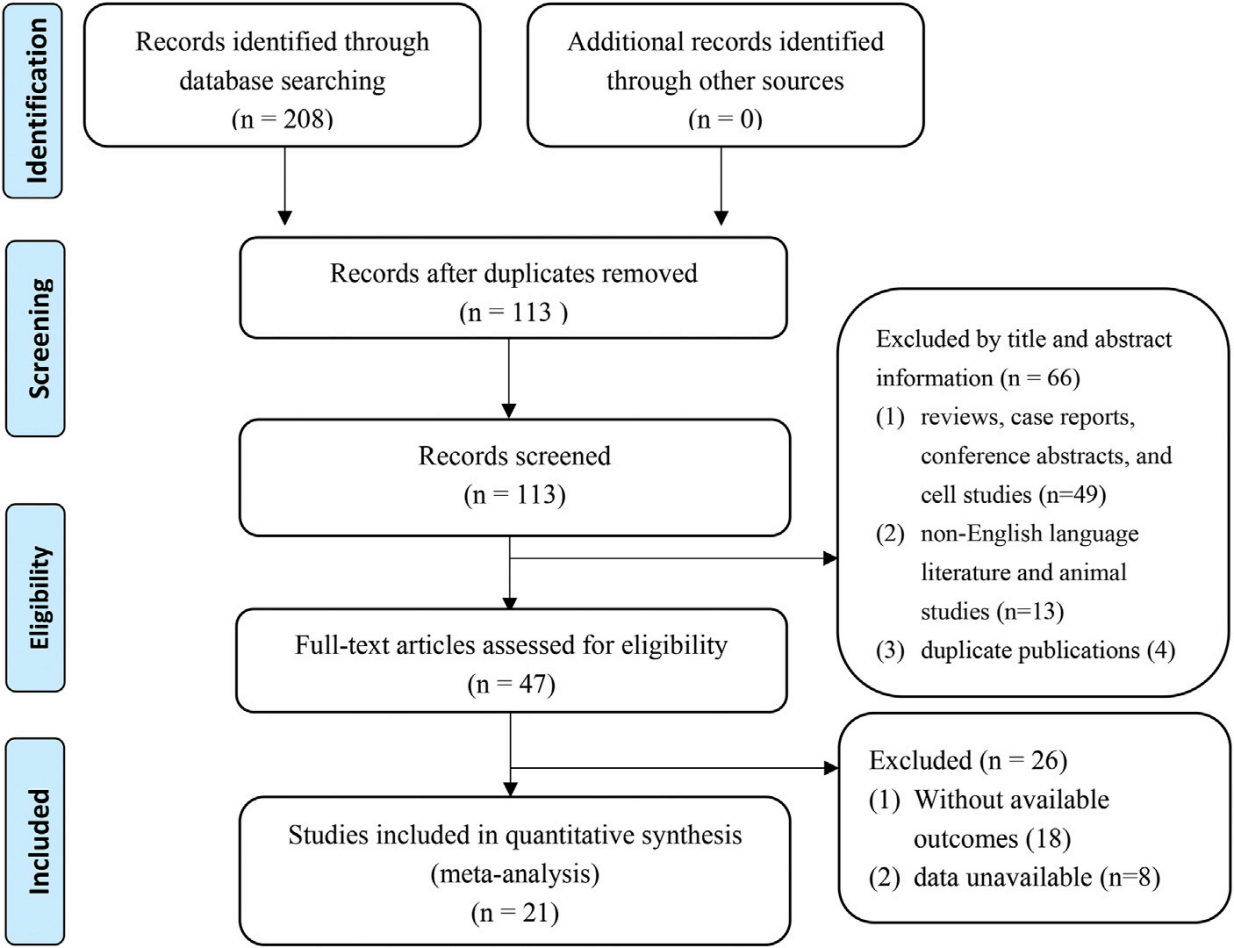
Flow diagram of this meta-analysis.

A total of 208 studies were identified after removing duplication. One hundred thirteen studies required further screening based on their title and abstract. Subsequently, 47 studies were eligible to read the full text, and eventually, 21 cohort studies (Zhang Y. Y. et al., 2019; Zhang Z. et al., 2019; Deng et al., 2019; Ji et al., 2019; Li et al., 2019; Pei et al., 2019; Zhao et al., 2019; Zhao and Huang, 2019; Zhang H. et al., 2020; Zhang K. et al., 2020; Chen et al., 2020; Zhang W. et al., 2020; Luo et al., 2020; Sun et al., 2020; Wang and Wen, 2020; Xu et al., 2020; Zhou et al., 2020; Wang et al., 2021a; Wang et al., 2021b; Feng et al., 2021; Liao et al., 2021) were included in the meta-analysis (Figure 1).

### 3.2 Studies characteristics and quality assessment

Twenty-one studies involving 1,080 patients were from China. The sample sizes ranged from 24 to 99. The recruited studies had 11 cancer types, including colorectal cancer, hepatocellular carcinoma, bladder cancer, non-small cell lung carcinoma, endometrial carcinoma, acute myeloid leukemia, retinoblastoma, prostate cancer, cervical cancer, pancreatic cancer, and ovarian cancer. The expression of SNHG14 was detected using qPCR within the included studies. In addition, 16 studies (Zhang Y. Y. et al., 2019; Zhang Z. et al., 2019; Ji et al., 2019; Li et al., 2019; Pei et al., 2019; Zhao et al., 2019; Zhao and Huang, 2019; Zhang H. et al., 2020; Zhang K. et al., 2020; Chen et al., 2020; Zhang W. et al., 2020; Luo et al., 2020; Sun et al., 2020; Wang and Wen, 2020; Feng et al., 2021; Liao et al., 2021) reported on OS, and 17 (Zhang Y. Y. et al., 2019; Zhang Z. et al., 2019; Deng et al., 2019; Ji et al., 2019; Zhang H. et al., 2020; Zhang K. et al., 2020; Chen et al., 2020; Zhang W. et al., 2020; Luo et al., 2020; Sun et al., 2020; Wang and Wen, 2020; Xu et al., 2020; Zhou et al., 2020; Wang et al., 2021a; Wang et al., 2021b; Feng et al., 2021; Liao et al., 2021) on clinical outcomes.

The NOS scores of included studies were as follows: two studies (Zhang Z. et al., 2019; Zhang W. et al., 2020) with a NOS score of 8, five studies (Ji et al., 2019; Zhang K. et al., 2020; Wang et al., 2021a; Wang et al., 2021b; Feng et al., 2021) with a NOS score of 7, eight studies (Zhang Y. Y. et al., 2019; Deng et al., 2019; Pei et al., 2019; Chen et al., 2020; Luo et al., 2020; Wang and Wen, 2020; Xu et al., 2020; Liao et al., 2021) with a NOS score of 6 and six studies (Li et al., 2019; Zhao et al., 2019; Zhao and Huang, 2019; Zhang W. et al., 2020; Sun et al., 2020; Zhou et al., 2020) with a NOS score of 5. The details are provided in Table 1.

**TABLE 1:** Characteristics of included studies.

Study	Year	Country	Cancer type	Sample type	Sample size	Detection method	Cut-off value	Outcome measure	Hazard ratios	Follow-up (month)	NOS score
Wang et al. (Wang et al., 2021b)	2021	China	Colorectal cancer	Tissue	30	qRT-PCR	Mean	CP	NA	NA	7
Liao et al. (Liao et al., 2021)	2021	China	Hepatocellular carcinoma	Tissue	66	qRT-PCR	Median	OS CP	K-M curve	80	6
Feng et al. (Feng et al., 2021)	2021	China	Bladder cancer	Tissue	62	qRT-PCR	Median	OS CP	K-M curve	36	7
Wang et al. (Wang et al., 2021a)	2021	China	Acute myeloid leukaemia	Bone marrow	57	qRT-PCR	Mean	CP	NA	NA	7
Zhou et al. (Zhou et al., 2020)	2020	China	Non-small cell lung cancer	Tissue	30	qRT-PCR	Mean	CP	NA	NA	5
Chen et al. (Chen et al., 2020)	2020	China	Non-small cell lung cancer	Tissue	50	qRT-PCR	NA	OS CP	K-M curve	60	6
Zhang et al. (Zhang et al., 2020c)	2020	China	Colorectal cancer	Tissue	92	qRT-PCR	Median	OS CP	K-M curve	60	8
Zhang et al. (Zhang et al., 2020b)	2020	China	Endometrial Carcinoma	Tissue	53	qRT-PCR	Mean	OS CP	K-M curve	45	7
Zhang et al. (Zhang et al., 2020a)	2020	China	Hepatocellular carcinoma	Tissue	40	qRT-PCR	Median	OS CP	K-M curve	36	5
Xu et al. (Xu et al., 2020)	2020	China	Hepatocellular carcinoma	Tissue	55	qRT-PCR	Median	CP	NA	NA	6
Sun et al. (Sun et al., 2020)	2020	China	Retinoblastoma	Tissue	43	qRT-PCR	Mean	OS CP	K-M curve	60	5
Wang et al. (Wang and Wen, 2020)	2020	China	Endometrial Carcinoma	Tissue	52	qRT-PCR	Mean	OS CP	K-M curve	72	6
Luo et al. (Luo et al., 2020)	2020	China	Prostate cancer	Tissue	60	qRT-PCR	Median	OS CP	K-M curve	60	6
Zhang et al. (Zhang et al., 2019b)	2019	China	Non-small cell lung cancer	Tissue	99	qRT-PCR	Median	OS CP	K-M curve	60	8
Ji et al. (Ji et al., 2019)	2019	China	Cervical cancer	Tissue	80	qRT-PCR	Mean	OS CP	K-M curve	60	7
Deng et al. (Deng et al., 2019)	2019	China	Pancreatic cancer	Tissue	45	qRT-PCR	Median	CP	NA	NA	6
Zhao et al. (Zhao and Huang, 2019)	2019	China	Ovarian cancer	Tissue	24	qRT-PCR	Median	OS	K-M curve	72	5
Zhao et al. (Zhao et al., 2019)	2019	China	Ovarian cancer	Tissue	56	qRT-PCR	Median	OS	K-M curve	60	5
Zhang et al. (Zhang et al., 2019a)	2019	China	Cervical cancer	Tissue	30	qRT-PCR	Median	OS CP	K-M curve	80	6
Pei et al. (Pei et al., 2019)	2019	China	Colorectal cancer	Tissue	32	qRT-PCR	Mean	OS	K-M curve	60	6
Li et al. (Li et al., 2019)	2019	China	Bladder cancer	Tissue	24	qRT-PCR	Mean	OS	K-M curve	60	5

OS: overall survival; CP: clinicopathological; NA: not reported.

### 3.3 Association between the expression level of SNHG14 and OS

**FIGURE 2:**
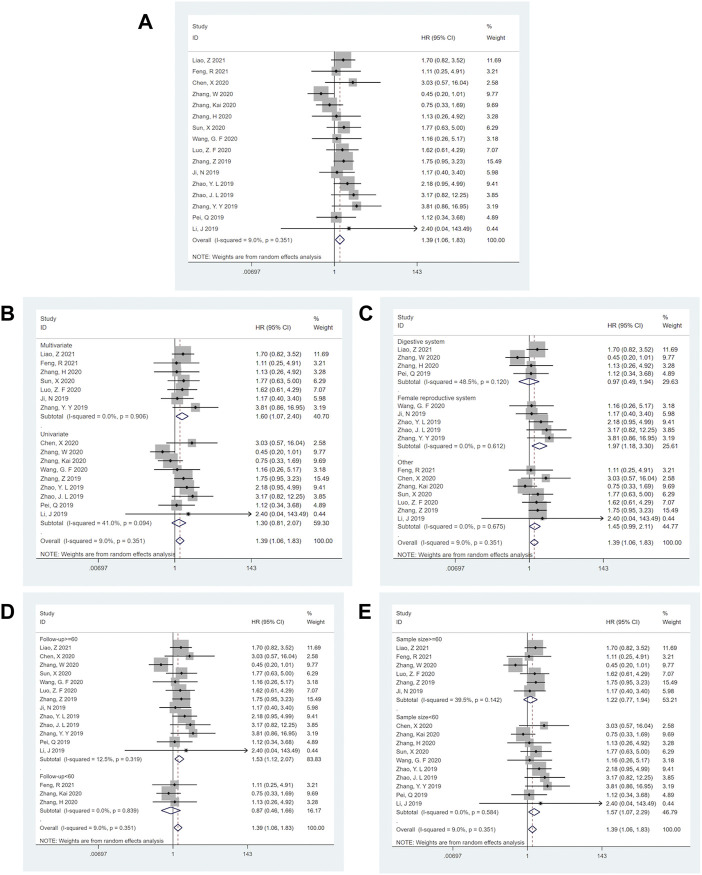
Forest plots for the association of SNHG14 expression with overall survival and subgroup analysis of SNHG14 expression with overall survival. **(A)** Forest plots for the association of SNHG14 expression with overall survival. **(B)** Subgroup analysis stratified by analysis method. **(C)** Subgroup analysis stratified by cancer type. **(D)** Subgroup analysis stratified by follow-up time. **(E)** Subgroup analysis stratified by sample size.

Sixteen studies involving 1,578 patients reported OS among cancer patients. Our meta-analysis depicted a statistically significant difference (HR = 1.39; 95%CI: (1.06–1.83); *p* = 0.017) (Figure 2A) and observed low heterogeneity (*I* ^2^ = 9.0%, *p* = 0.35), fixed-effects model was used. In the high SNHG14 expression group, patients with low survival rates significantly increased, indicating that SNHG14 is an independent factor in the survival of patients having malignant tumors.

Subgroup analysis demonstrated that the high expression SNHG14 during multivariate analysis [HR = 1.60; 95% CI: (1.07–2.40); *p* = 0.022] (Figure 2B), the female reproductive system cancer [HR = 1.97; 95% CI: (1.18–3.30); *p* = 0.009] (Figure 2C), the follow-up time of > =60 months [HR = 1.53; 95% CI: (1.12–2.07); *p* = 0.007] (Figure 2D) and the sample size of < 60 tissues [HR = 1.57; 95% CI: (1.07–2.29); *p* = 0.002] (Figure 2E) were statistically significant and related to low OS. These analyses are depicted in Table 2.

**TABLE 2:** Result of SNHG14 expression with overall survival.

Subgroup analysis		Patients (n)	HR (95%CI)	*p*-value	Heterogeneity (*I* ^2^)	Model
Follow-up	≥ 60 months	708	1.53 (1.12–2.07)	0.007	12.5%	Fixed
	< 60 months	155	0.87 (0.46–1.66)	0.677	0.0%	Fixed
Sample size	≥ 60	459	1.22 (0.77–1.94)	0.394	39.5%	Fixed
	<60	404	1.57 (1.07–2.29)	0.020	0.0%	Fixed
Analysis method	Multivariate	381	1.60 (1.07–2.40)	0.022	0.0%	Fixed
	Univariate	482	1.30 (0.81–2.07)	0.274	41.0%	Fixed
Cancer type	Digestive system	230	0.97 (0.49–1.94)	0.942	48.5%	Fixed
	Female reproductive system	242	1.97 (1.18–3.30)	0.009	0.0%	Fixed
	Other systems	391	1.49 (0.99–2.11)	0.055	0.0%	Fixed
Overall results		863	1.39 (1.06–1.83)	0.017	9.0%	Fixed

### 3.4 Association between SNHG14 and clinicopathological features

**FIGURE 3:**
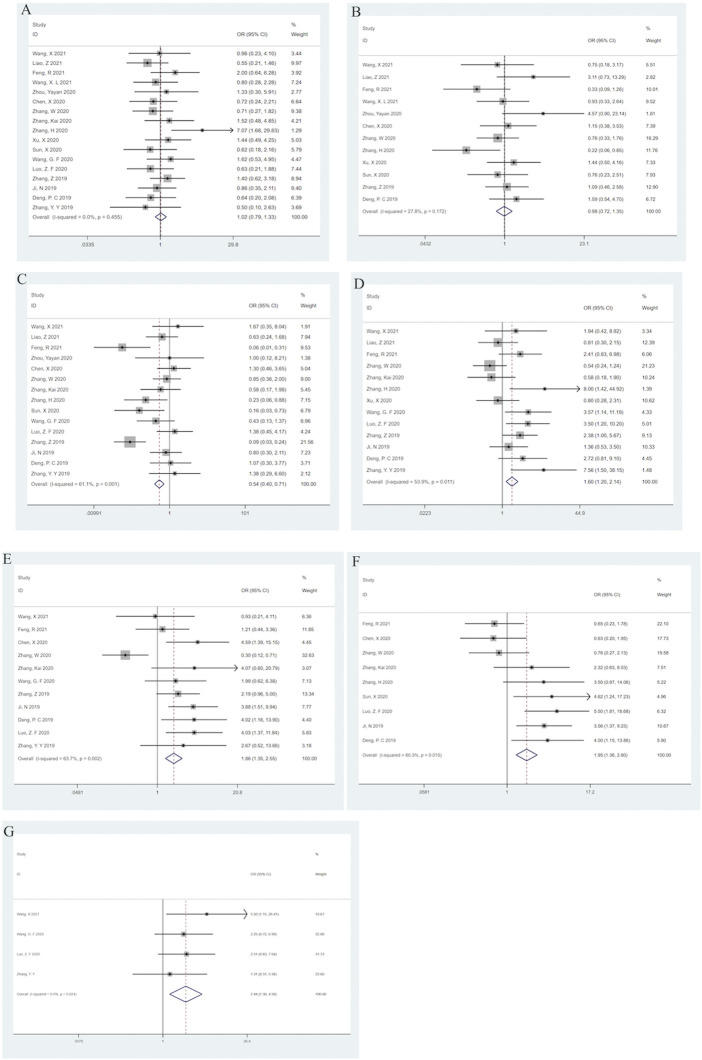
Forest plots for association of SNHG14 expression with clinicopathological features: Age **(A)**, Gender **(B)**, TNM staging **(C)**, Tumor size **(D)**, Lymph node metastasis **(E)**, Differentiation grade **(F)**, Distant metastasis **(G)**.

Seventeen reported a link between clinicopathological characteristics and SNHG14. High SNHG14 expression was observed to be significantly correlated with TNM Staging (II-III) (OR = 0.54; 95% CI: (0.40–0.71); *p* < 0.001) having high heterogeneity *I* ^2^ = 61.1%, *p* = 0.001) (Figure 3C), tumor size > 3.5 cm (OR = 1.60; 95% CI: (1.20–2.14); *p* = 0.001) showing high heterogeneity (*I* ^2^ = 53.9%, *p* = 0.011) (Figure 3D), lymph node metastasis [OR = 1.86; 95% CI: (1.35–2.55); *p* < 0.001] having high heterogeneity (*I* ^2^ = 63.7.9%, *p* = 0.002) (Figure 3E), low differentiation grade [OR = 1.95; 95% CI(1.36–2.80); *p* < 0.001] with significant heterogeneity (*I* ^2^ = 60.3%, *p* = 0.010) (Figure 3F), and distant metastasis [OR = 2.44; 95% CI: (1.30–4.58); *p* = 0.005] without any heterogeneity (*I* ^2^ = 0.0%, *p* = 0.624) (Figure 3G). However, the meta-analysis revealed that there was no significant correlation between SNHG14 expression and age [OR = 0.98; 95% CI: (0.72–1.35); *p* = 0.915] without any heterogeneity (*I* ^2^ = 0.0%, *p* = 0.455) (Figure 3A) or gender [OR = 0.98; 95% CI: (0.72–1.35); *p* = 0.915] having low heterogeneity (*I* ^2^ = 27.8%, *p* = 0.172) (Figure 3B). These analyses are represented in Figure 3; Table 3.

**TABLE 3:** Result of SNHG14 expression with clinicopathological feature.

Clinicopathological parameters studies (*n*)	Patients (n)	Or (95% CI)	*p*-value	Heterogeneity (*I* ^2^)	Model	Begg’s test (*p*)	Egger’s test (*p*)
Age (elderly vs. non-elderly)	944	1.02 (0.77–1.33)	0.863	0.0%	Fixed	0.934	0.538
Gender (male vs. female)	679	0.98 (0.72–1.35)	0.915	27.8%	Fixed	0.493	0.756
Tumor size (large size vs. small size)	764	1.60 (1.20–2.14)	0.001	53.9%	Random	0.174	0.405
TNM stage (III + IV vs. I + II)	820	0.54 (0.40–0.71)	<0.001	61.1%	Random	0.586	0.723
Lymph node metastasis (positive vs. negative)	653	1.86 (1.35–2.55)	<0.001	63.7%	Random	0.938	0.349
Differentiation grade (poorly vs. moderately and well)	525	1.95 (1.36–2.80)	<0.001	60.3%	Random	0.532	0.335
Distant metastasis (presence vs. absence)	172	2.44 (1.30–4.58)	0.005	0.0%	Fixed	1.000	0.743

### 3.5 Publication bias and sensitivity analysis

**FIGURE 4:**
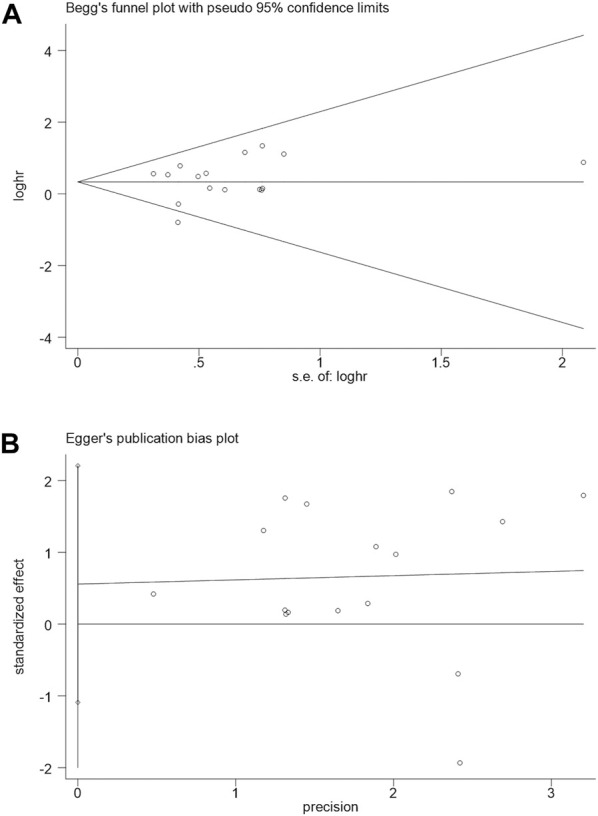
Begg’s publication bias plots**(A)** and Egger funnel regression test**(B)** of overall survival.

**FIGURE 5:**
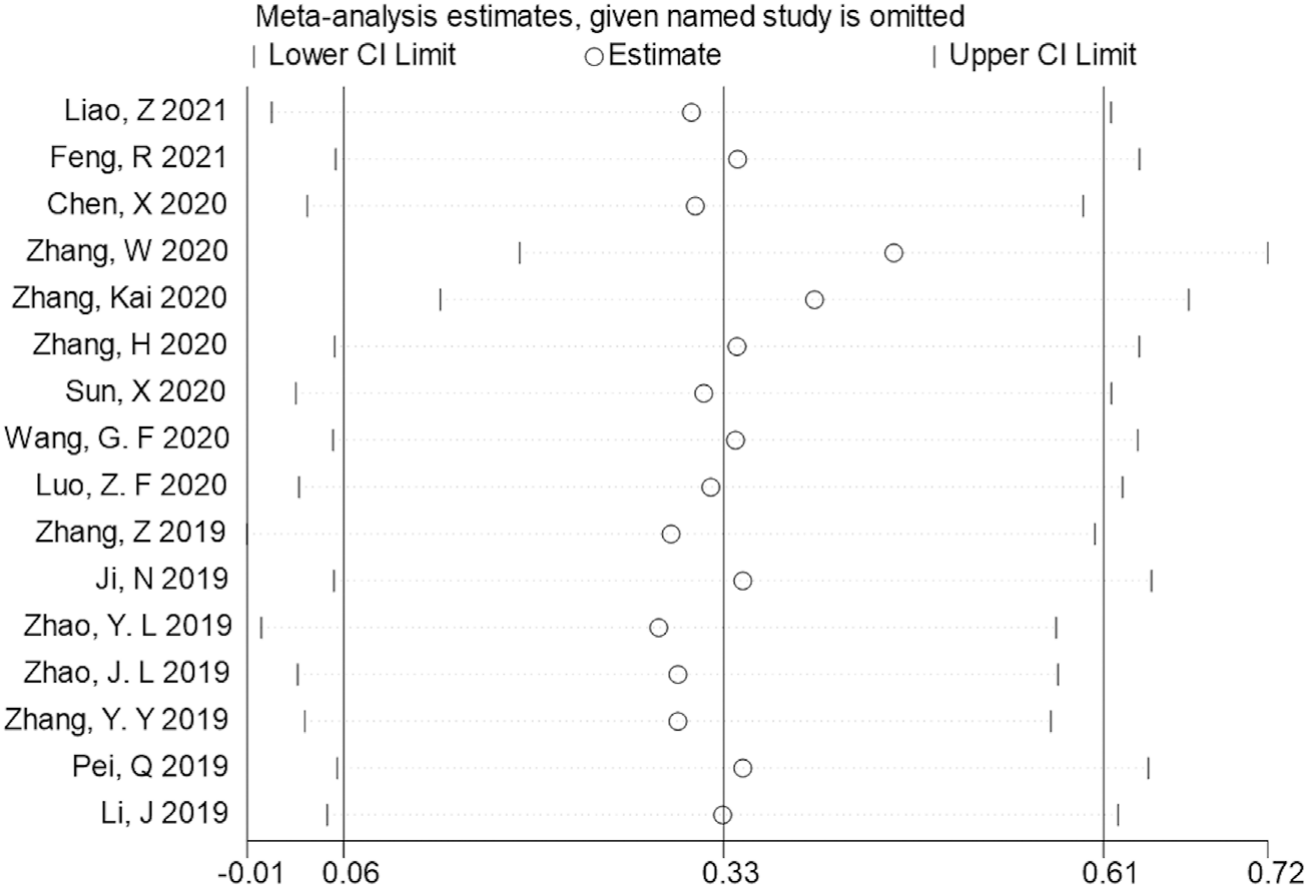
Sensitivity analysis for studies about OS by omitting each study sequentially.

Begg’s and Egger’s regression tests were utilized to explore the publication bias of the studies in our meta-analysis, and a funnel plot was created to determine the publication bias. No publication bias was observed [Begg funnel plot (Pr>|z| = 0.528) (Figure 4A) and Egger funnel plot (*p*>|t| = 0.480) (Figure 4B)], suggesting that our pooled results were credible. In addition, a sensitivity analysis explored their potential source and assessed the robustness of these outcomes. After omitting each included study in turn for each outcome, the results of OS remained stable. Therefore, the predicted aggregated results of the OS based on SNHG14 expression were reliable (Figure 5).

### 3.6 Result of trial sequential analysis

**FIGURE 6:**
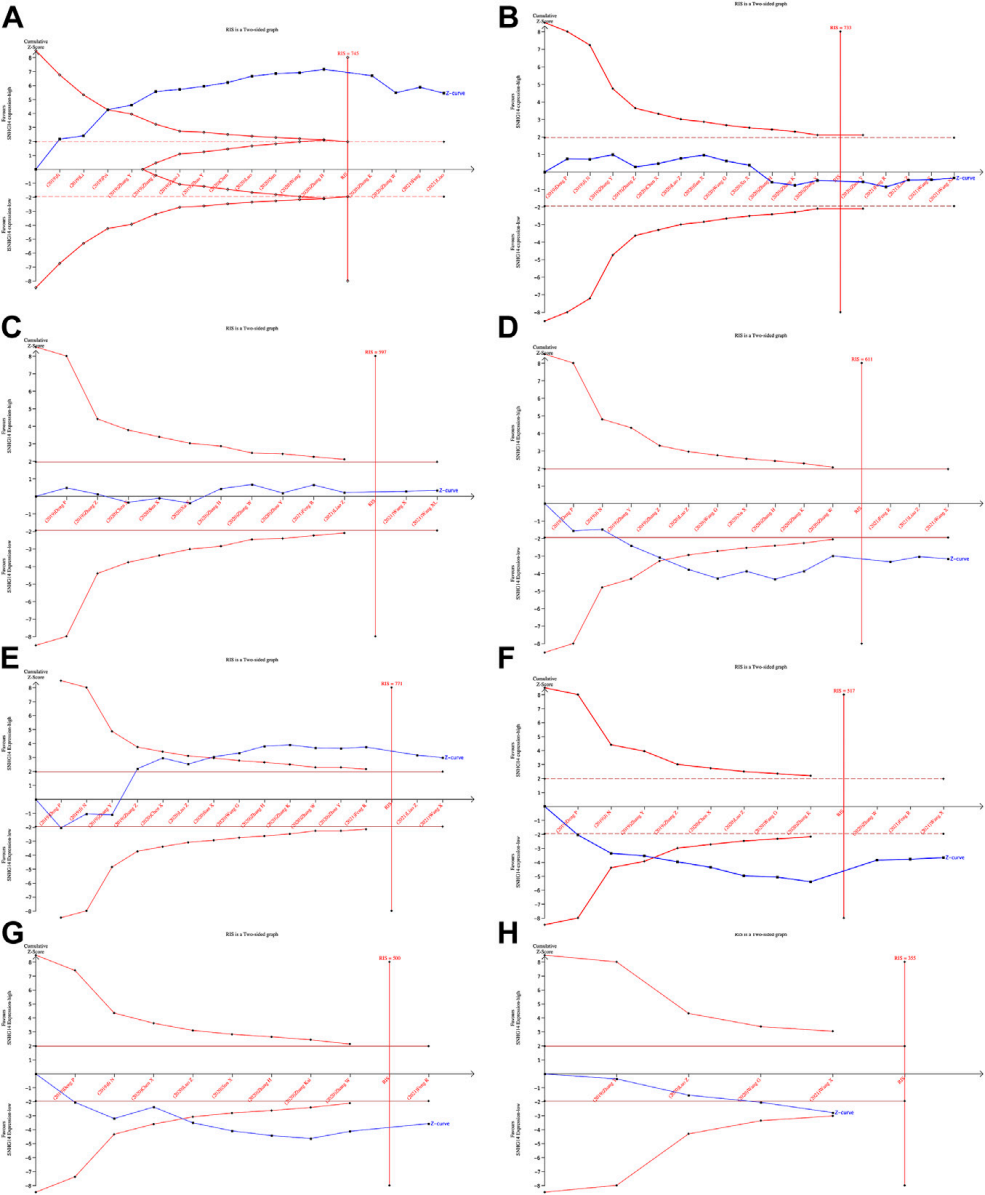
Results of TSA:OS**(A)**, Age**(B)**, Gender**(C)**, Tumor size **(D)**, TNM staging**(E)**, Lymph node metastasis**(F)**, Differentiation grade**(G)**, Distant metastasis**(H)**.

The TSA of OS, tumor size, TNM staging, Lymph node metastasis, and Differentiation grade showed that the Z-curve crossed the conventional boundary and RIS, indicating robust evidence. The TSA of age and gender grade showed that the Z-curve crossed the RIS but not the conventional boundary, which stated that false positive conclusions might be obtained. However, The TSA for the distant metastasis revealed that the Z-curve did not cross the conventional or trial sequential monitoring boundary and the RIS (= 355). Therefore, the evidence on the effect of distant metastasis was insufficient. (Figure 6).

### 3.7 Analysis of SNHG14-Related genes

**FIGURE 7:**
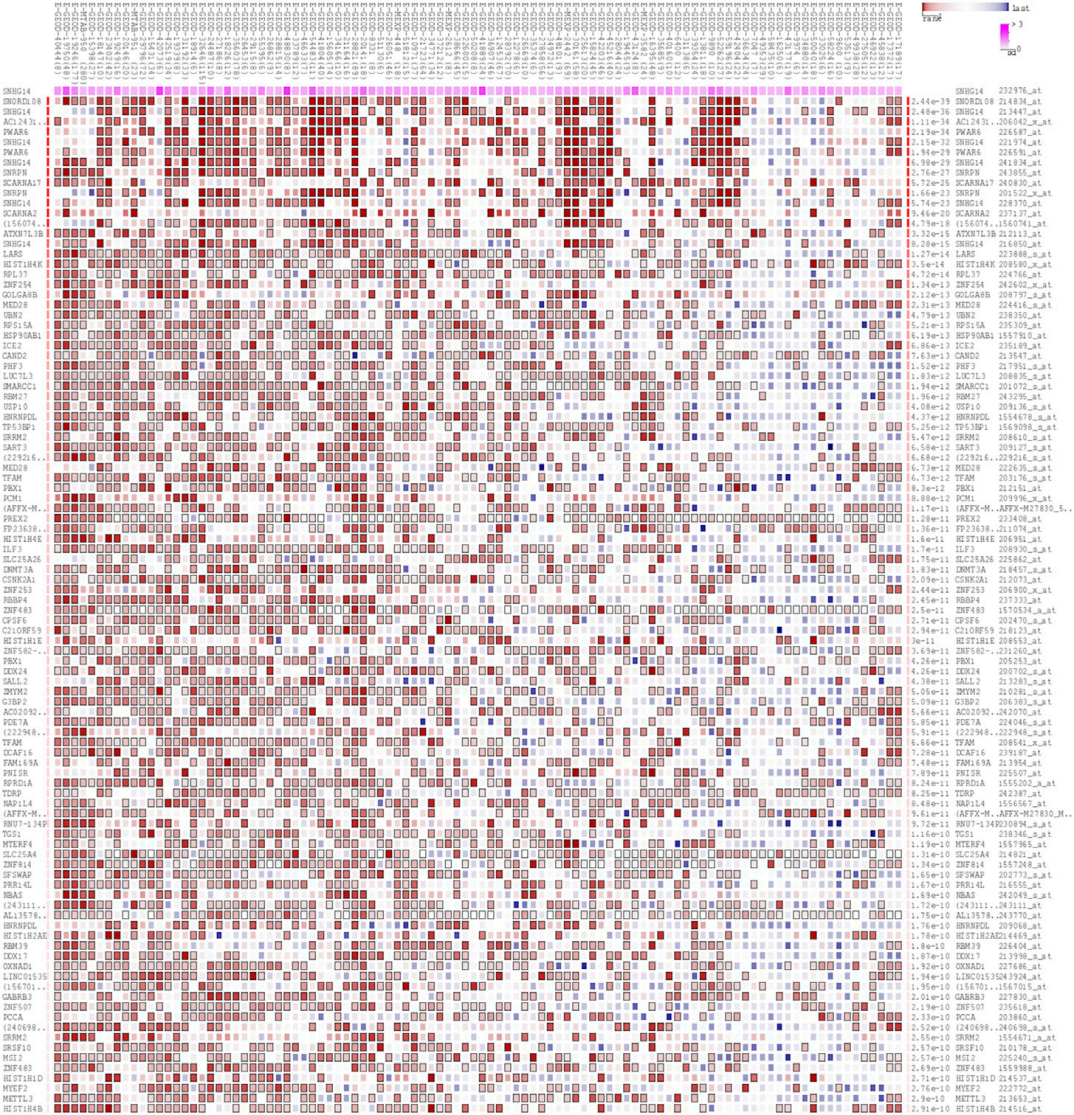
The top 150 predicted target genes of SNHG14 by using Multi Experiment Matrix (MEM, http://biit.cs.ut.ee/mem/) website.

**FIGURE 8:**
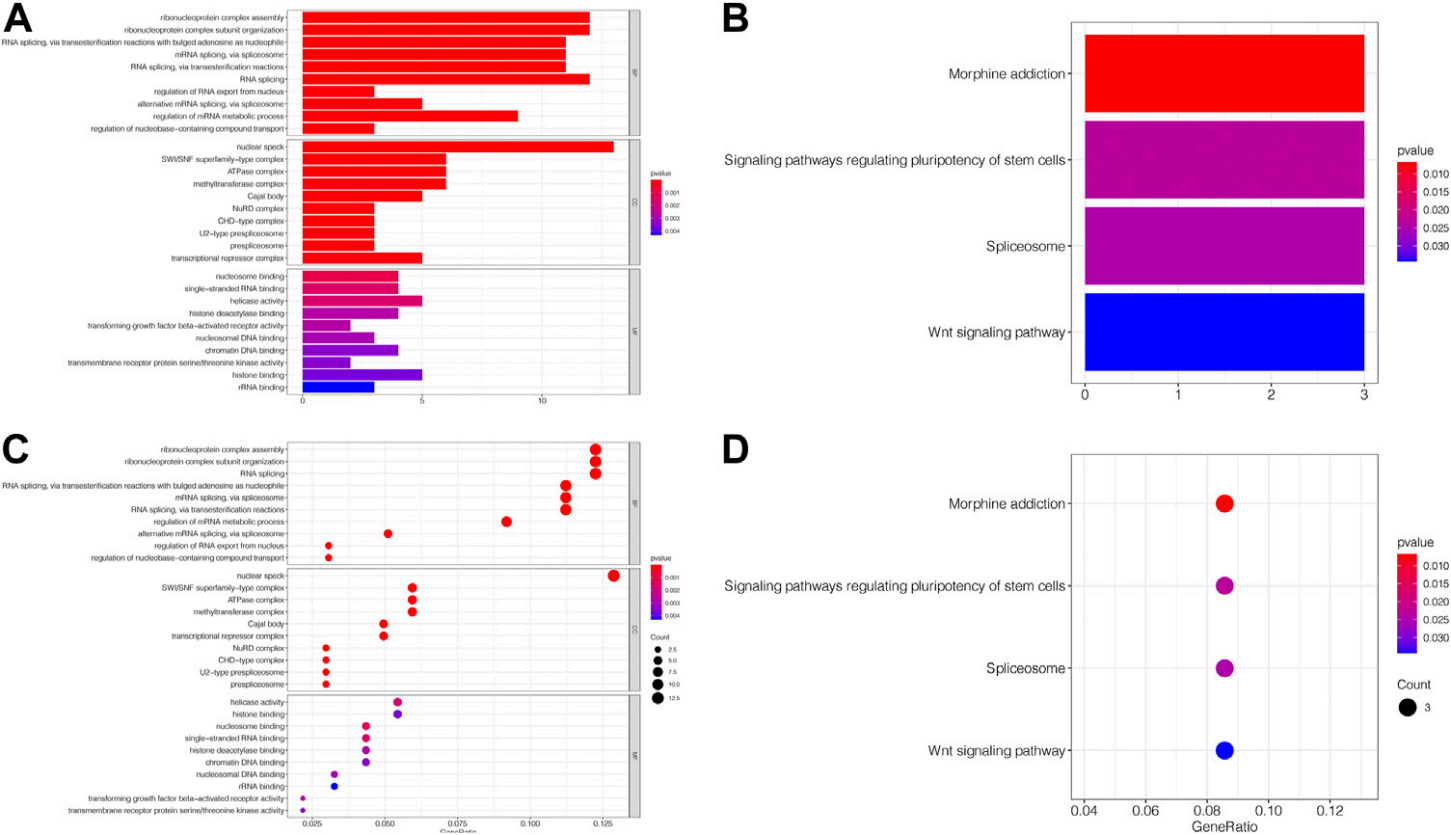
GO terms and the KEGG pathway. **(A)** Histogram presentation of the top 10 positions of GO terms of target genes in biological processes (BP), cellular components (CC) and molecular functions (MF) of biology; **(B)** Histogram presentation of pathways related to the differentially expressed genes by the KEGG analysis; **(C)** Bubble chart of the top 10 positions of GO terms of target genes in BP, CC, MF; **(D)** Bubble chart of pathways related to the differentially expressed genes by the KEGG analysis.

**FIGURE 9:**
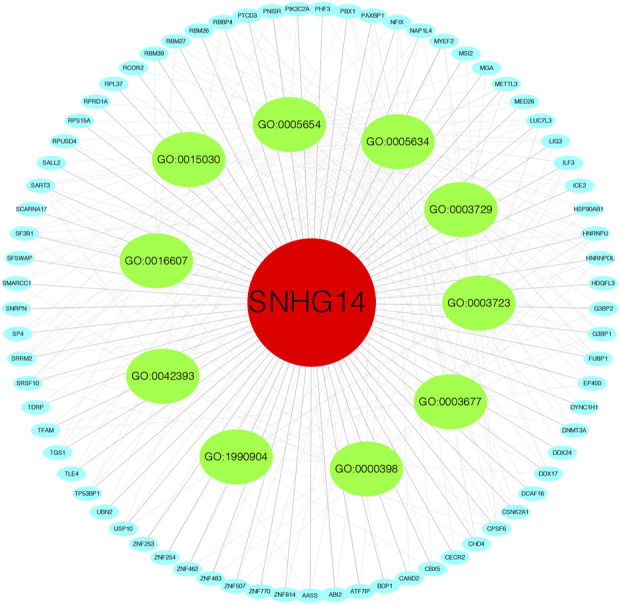
Differentially expressed gene interaction network analysis.

The MEM database was utilized to screen the top 150 co-expressed genes of SNHG14. SNORD108, AC124312.1, and PWAR6 were the top three target genes ranked by *p*-value, significantly associated with SNHG14 gene expression (Figure 7). GO and KEGG pathway analyses were performed to explore the underlying molecular mechanisms. The results of GO analysis depicted those co-expressed genes primarily involved in biological processes (BP), such as the ribonucleoprotein complex assembly, ribonucleoprotein complex subunit organization, and RNA splicing; cellular components (CC), including nuclear speck, ATPase complex, and SWI/SNF superfamily-type complex; molecular function (MF), involving helicase activity, nucleosome binding, and single−stranded RNA binding (Figure 7). In addition, the KEGG pathway analysis revealed that co-expressed genes were implicated in Morphine addiction, Spliceosome, and Wnt signaling pathway (Figure 8). Moreover, a signal pathway network was developed using the Cytoscape software (Figure 9; Table 4).

**TABLE 4:** Summary of FOXP4-AS1 functional roles and related genes.

GO number	Description	Genes	*p* value	FDR
GO:0003723	RNA binding	RBM27, RBM26, HSP90AB1, DDX24, USP10, SFSWAP, HNRNPU, MSI2, RPS15A, SART3, HNRNPDL, G3BP1, G3BP2, RPL37, SRSF10, SF3B1, DDX17, SRRM2, PTCD3, PNISR, RBM39, DYNC1H1, CPSF6, SNRPN, MYEF2, NAP1L4, RPUSD4, ILF3, FUBP1, LUC7L3, TFAM	3.32E-12	6.93E-10
GO:0005654	nucleoplasm	NFIX, HSP90AB1, USP10, ICE2, HNRNPU, CHD4, PIK3C2A, DCAF16, RPS15A, SART3, HNRNPDL, RBBP4, UBN2, TGS1, EP400, RPRD1A, TP53BP1, SRSF10, SF3B1, DDX17, SRRM2, ATF7IP, TLE4, PTCD3, PNISR, RBM39, SMARCC1, CPSF6, CBX5, SNRPN, CSNK2A1, MGA, METTL3, DNMT3A, LIG3, PBX1, MED28, RPUSD4, ILF3, ABI2, SP4, FUBP1, LUC7L3, BDP1, HDGFL3	7.01E-09	1.34E-06
GO:0005634	nucleus	RBM27, PHF3, ZNF254, ZNF253, ZNF770, RBM26, HSP90AB1, HNRNPU, TDRP, CHD4, SART3, SALL2, AASS, DDX17, TLE4, SRRM2, ZNF483, SMARCC1, CSNK2A1, METTL3, DNMT3A, LIG3, MED28, ILF3, LUC7L3, TFAM, HDGFL3, NFIX, USP10, SFSWAP, CAND2, CECR2, HNRNPDL, RBBP4, ZNF507, UBN2, TGS1, G3BP1, EP400, TP53BP1, SRSF10, SF3B1, ATF7IP, ZNF462, RBM39, CPSF6, CBX5, MYEF2, PBX1, NAP1L4, ABI2, SP4, FUBP1, ZNF814, PAXBP1, RCOR2	2.88E-08	2.75E-06
GO:0016607	nuclear speck	SRRM2, RBM27, DDX17, PNISR, RBM39, CPSF6, METTL3, HNRNPU, SART3, EP400, LUC7L3, SRSF10, SF3B1	1.00E-06	6.39E-05
GO:1990904	ribonucleoprotein complex	DDX17, ILF3, MYEF2, CPSF6, CBX5, G3BP1, G3BP2, HNRNPU	1.52E-05	7.26E-04
GO:0003729	mRNA binding	SRRM2, MYEF2, CPSF6, FUBP1, G3BP1, METTL3, G3BP2, LUC7L3, SF3B1	1.76E-05	0.001834
GO:0042393	histone binding	SMARCC1, SART3, RBBP4, CHD4, TP53BP1, AASS, NAP1L4	2.66E-04	0.018525
GO:0015030	Cajal body	SRRM2, SART3, ICE2, TGS1, SCARNA17	5.09E-04	0.019437
GO:0003677	DNA binding	ZNF462, ZNF483, NFIX, MGA, DNMT3A, HNRNPU, LIG3, CHD4, PBX1, ILF3, HNRNPDL, ZNF507, G3BP1, EP400, LUC7L3, TFAM, PAXBP1	4.04E-04	0.021134
GO:0000398	mRNA splicing, via spliceosome	SRRM2, SART3, SNRPN, METTL3, HNRNPU, PAXBP1, SRSF10, SF3B1	5.46E-05	0.030088

### 3.8 The relationship between SNHG14 expression in cancer and TMB and MSI

**FIGURE 10:**
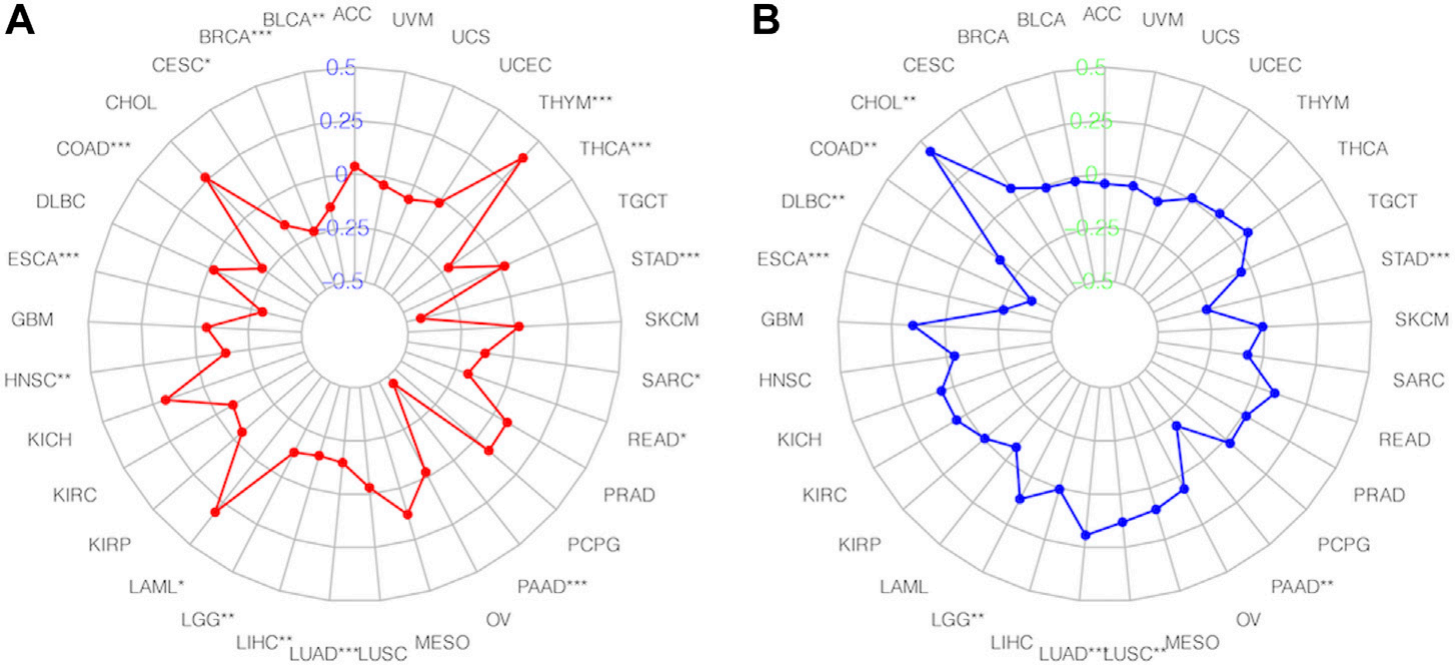
Relationships between SNHG14 gene expression and TMB**(A)**, MSI, **(B)** in different types of cancers.

TMB and MSI are essential determinants in tumor incidence and progression. Thus, we evaluated the association between SNHG14 expression and TMB or MSI to assess its immunogenicity (Chalmers et al., 2017). Our findings described that SNHG14 expression was positively related to TMB in colon adenocarcinoma (COAD), thymoma (THYM), and acute myeloid leukemia (LAML), However, the SNHG14 expression was negatively associated with TMB in 11 cancers, including esophageal carcinoma (ESCA), lung adenocarcinoma (LUAD), PAAD, stomach adenocarcinoma (STAD), thyroid carcinoma (THCA), bladder urothelial carcinoma (BLCA), head and neck squamous cell carcinoma (HNSC), LGG, LIHC, and rectum adenocarcinoma (READ) (Figure 10A).

Moreover, we investigated the association of SNHG14 expression with MSI in specific cancers. Our findings showed that SNHG14 expression was strongly related to MSI in nine cancer types, with four (LUAD, cholangiocarcinoma (CHOL), LGG, and lung squamous cell carcinoma (LUSC)) positively associated with MSI. However, in ESCA, STAD, COAD, lymphoid neoplasm diffuse large B-cell lymphoma (DLBC), and PAAD, the SNHG14 expression was negatively associated with MSI (Figure 10B).

### 3.9 Correlation of SNHG14 expression in cancer with immune cell infiltration

**FIGURE 11:**
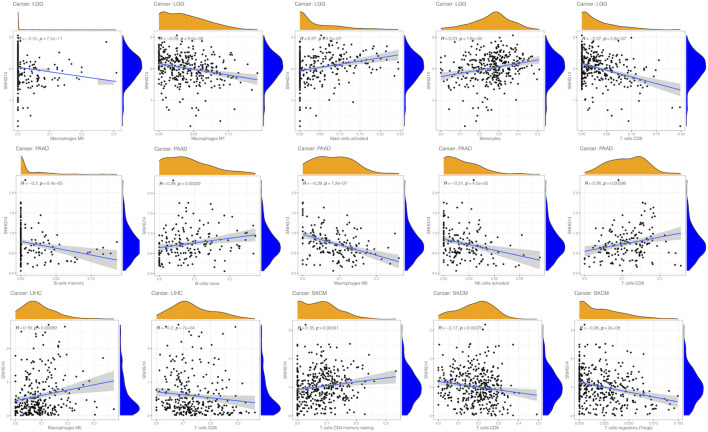
Relationship between SNHG14 gene expression and infiltrating levels of immune cells in LGG, PAAD, MESO, SKCM, LIHC.

Our findings revealed that SNHG14 expression was positively related to infiltrated active mast cells and monocytes in LGG. However, it was negatively correlated with infiltrating M0 macrophages, M1 macrophages, and CD8 T cells within PAAD. Moreover, SNHG14 expression was positively related to naïve B cells and CD8 T cells infiltrated but negatively correlated with memory B cells, M0 macrophages, and activated NK cells infiltrated inside PAAD. The expression of SNHG14 in LIHC was positively associated with infiltrating M0 macrophages and negatively correlated with infiltrating CD8 T cells in LIHC. In SKCM, the SNHG14 expression was positively related to resting memory CD4 T cells and regulatory T cells (Tregs) but negatively associated with CD8 T cells (Figure 11). Thus, SNHG14 may be involved in the immune infiltration of M0 macrophages and could cause cancer by affecting the tumor microenvironment.

## 4 Discussion

Cancer poses a severe threat to human health. Despite significant cancer detection and treatment advancements, cancer incidence has gradually increased in recent years (Jiang and Le, 2020). Many studies have indicated that lncRNA is dysregulated in cancer and vital to tumor development and progression. For instance, a meta-analysis by Zhou et al. (2018) showed that overexpression of lncRNA-XIST is correlated with poor prognosis and clinicopathological characteristics. LncRNA-XIST could be a promising non-invasive biomarker for determining prognosis and clinical pathology.

SNHG14 is highly expressed in various cancers and is reported to accelerate tumor development (Xu et al., 2019). SNHG14 is upregulated within tumor tissues, including colorectal cancer, hepatocellular carcinoma, bladder cancer, non-small cell lung carcinoma, endometrial carcinoma, acute myeloid leukemia, retinoblastoma, prostate cancer, cervical cancer, pancreatic cancer, and ovarian cancer. SNHG14 participates in reprogramming glucose metabolism and tumorigenesis within gliomas by interacting using the RNA-binding protein Lin28A. Silencing SNHG14 inhibits glycolysis and proliferation of glioma cells while enhancing cell apoptosis (Lu et al., 2020). In contrast, another study demonstrated the role of SNHG14 in inhibiting glioma cell proliferation, invasion and promoting apoptosis. The role of SNHG14 in different cancers is still controversial (Wang et al., 2018). Therefore, this meta-analysis was undertaken to investigate the relationship between the expression of SNHG14 and clinicopathological features and patient prognosis.

According to the results of the meta-analysis, high expression of the SNHG14 gene is associated with a poor prognosis. The overall results revealed that high SNHG14 expression is associated with poor tumor prognosis. Increased SNHG14 expression was observed to associate with tumor size (> 3.5 cm), TNM staging (II-III), lymph node metastasis, low differentiation grade, and distant metastasis but not with age and gender. Although data have revealed SNHG14 as an important prognostic factor for different types of tumors, the molecular mechanism behind how it affects cancer is unknown. SNHG14 enhances the progression of DLBCL by isolating miR-152-3p, preventing it from inhibiting the PD-1/PD-L1 checkpoint (Tian et al., 2021). The LncRNA SNHG14 is upregulated and activated with SP1 regulators in ccRCC cells. SNHG14 can promote renal cancer cell migration and invasion through sponge miR-203 and release N-WASP as ceRNA (Liu et al., 2017). Furthermore, the transfer of lncRNA SNHG14 mediated using exosomes induces breast cancer cell resistance to trastuzumab. Moreover, exosomal lncRNA SNHG14 within human serum is a potential breast cancer diagnostic biomarker enhancing the clinical benefit of trastuzumab therapy. To further explore the relationship between SNHG14 and other cancers, SNHG14 and its functions and related genes have been summarized in Table 5.

**TABLE 5:** Summary of lncRNA SNHG14 functional role and related gene.

Cancer type	Expression	Functional role	Related gene	Study
Colorectal cancer	Upregulate/downregulate	Cell proliferation, apoptosis, invasion migration, metastasis, EMT	miR-519b-3p/DDX5, miR-92b-3p, miR-944/KRAS	Wang (Wang et al., 2021b), Zhang (Zhang et al., 2020c) Pei (Pei et al., 2019)
Hepatocellular carcinoma	Upregulate	Cell proliferation, apoptosis, invasion migration, metastasis	miR-876-5p/SSR2, PABPC1, miR-217	Liao (Liao et al., 2021), Zhang (Zhang et al., 2020a) Xu (Xu et al., 2020)
Bladder cancer	Upregulate	Cell proliferation, apoptosis, invasion migration	miR-211-3p/ESM1, miR-150-5p	Feng (Feng et al., 2021), Li (Li et al., 2019)
Non-small cell lung cancer	Upregulate	Cell proliferation, apoptosis, invasion migration, metastasis	miR-382-5p, miR-340	Zhou (Zhou et al., 2020), Chen (Chen et al., 2020) Zhang (Zhang et al., 2019b)
Endometrial Carcinoma	Upregulate/downregulate	Cell proliferation, apoptosis, invasion migration, metastasis	miR-93-5p/ZBTB7A, miR-655-3p	Zhang (Zhang et al., 2020b), Wang (Wang and Wen, 2020)
Acute myeloid leukaemia	Upregulate	Cell proliferation, apoptosis	miR-193b-3p/MCL1	Wang (Wang et al., 2021a)
Retinoblastoma	Upregulate	Cell proliferation, apoptosis, invasion migration	miR-124	Sun (Sun et al., 2020)
Prostate cancer	Upregulate	Cell proliferation, apoptosis, invasion	miR-5590-3p/YY1	Luo(Luo et al., 2020)
Cervical cancer	Upregulate	Cell proliferation, apoptosis, invasion migration, EMT	miR-206/YWHAZ	Ji (Ji et al., 2019)
Pancreatic cancer	Upregulate	Cell proliferation, apoptosis, invasion	miR-613	Deng (Deng et al., 2019)
Ovarian cancer	Upregulate	Cell proliferation,, invasion migration	miR-125a-5p, DGCR8	Zhao (Zhao and Huang, 2019), Zhao (Zhao et al., 2019)

The target genes of SNHG14 were predicted and functionally annotated. SNORD108, AC124312.1, and PWAR6 were significantly co-expressed with SNHG14. GO and KEGG analysis revealed that co-expressed genes were involved in essential cell signaling pathways and physiological processes. SNHG14 functioned as a competing endogenous RNA for microRNAs-382-5p (miR-382-5p) to regulate the SPIN1 expression in non-small cell lung cancer (Chen et al., 2020). Another finding by bioinformatics analysis is that SNHG14 expression could be associated with TMB in 14 cancer types and MSI in nine cancer types. Robert’s research showed that TMB is associated with improved survival in patients receiving immune checkpoint inhibitors (ICI) across various cancer types (Samstein et al., 2019). Another finding by bioinformatics analysis is that SNHG14 expression may be related to TMB in 14 cancer types and MSI in 9 cancer types. Dynamic features of the TME, tumor-infiltrating cells, and immune biomarkers are critical for immunotherapy response (Kaderbhaï et al., 2019). Our study suggests that SNHG14 may play a vital role in the recruitment and regulation of immune-infiltrating cells in cancer, ultimately affecting the prognosis of patients.

This is the first study analyzing the relationship between the expression level of SNHG14 and the prognosis and clinical characteristics among cancer patients. Shen et al. (2021) previously described the expression profile, biological function, and molecular mechanism of SNHG14 in cancer, facilitating a molecular basis for future clinical application of SNHG14. We conducted a meta-analysis to evaluate the association between SNHG14 expression, OS, and the clinicopathological significance of different cancer types. This study included more original studies and detailed subgroup and sensitivity analyses compared to previous studies. There are several limitations to our research. First, the HR in the survival analysis was determined based on the KM curve from the literature, resulting in errors. Second, the patients included in the study were all China. Thus interpretation and application of the results require caution. Third, the individual differences of various cancer patients and their different lifestyles could also increase heterogeneity.

In conclusion, this meta-analysis demonstrated that high expression of the SNHG14 gene is associated with poor prognosis of East Asian cancer patients, predominantly female reproductive system cancer. Furthermore, differentially expressed SNHG14 could be used as an oncogene or cancer suppressor to improve cancer prognosis and identify potential therapeutic targets. In addition, well-designed studies with a larger sample size in different countries worldwide are expected to confirm our findings.

## Statement

“Clinicopathological significance and prognosis of long noncoding RNA SNHG14 expression in human cancers: A Meta-Analysis and bioinformatics analysis” (https://www.researchsquare.com/article/rs-1209386/v1) has been previously submitted in BMC Cancer. When we submitted it, the option to agree to publish a preprint was checked. However, the paper was rejected on 31 December 2021. According to the suggestions of reviewers, we have revised and removed several sections through discussion, which has been presented as the current version.

## Data Availability

The original contributions presented in the study are included in the article/Supplementary Material, further inquiries can be directed to the corresponding author.

## Abbreviations

lncRNA, Long noncoding RNA; ceRNA, Competing endogenous RNAs; SNHG14, small nucleolar RNA host gene 14; HR, Hazard ratio; OR, Odds ratio; OS, Overall survival; 95% CI, 95% Confidence interval; qRT-PCR, Quantitative real-time polymerase chain reaction; NOS, The Newcastle–Ottawa Quality Assessment Scale.
