# Supplementary material for: Overexpression of LncRNA SNHG14 as a biomarker of clinicopathological and prognosis value in human cancers: A meta-analysis and bioinformatics analysis
Source: Front Genet. 2022 Oct 6;13:945919. doi: 10.3389/fgene.2022.945919 (PMC9582150; doi:10.3389/fgene.2022.945919)
Supplement: Supplementary file 1 [file DataSheet1.docx]

**Supplementary file 1-Search strategy**

**PubMed**

**#1** "Neoplasms"[Mesh]

**#2** Tumor[Title/Abstract]) OR (Neoplasm[Title/Abstract])) OR (Tumors [Title/Abstract])) OR (Neoplasia[Title/Abstract])) OR (Neoplasias[Title/Abstract])) OR (Cancer[Title/Abstract])) OR (Cancers[Title/Abstract])) OR (Malignant Neoplasm [Title/Abstract])) OR (Malignancy[Title/Abstract])) OR (Malignancies[Title/Abstract])) OR (Malignant Neoplasms[Title/Abstract])) OR (Neoplasm, Malignant [Title/ Abstract])) OR (Neoplasms, Malignant [Title/Abstract])) OR (Benign Neoplasms [Title/Abstract])) OR (Benign Neoplasm [Title/Abstract])) OR (Neoplasms, Benign [Title/Abstract])) OR (Neoplasm, Benign[Title/Abstract])

**#3** **#1 OR #2**

**#4** SNHG14[Title/Abstract]) OR (small nucleolar RNA host gene 14[Title/Abstract])) OR (115HG[Title/Abstract])) OR (IC-SNURF- SNRPN [Title/Abstract])) OR (LNCAT [Title/Abstract])) OR (NCRNA00214 [Title/Abstract])) OR (U-UBE3A-ATS [Title/Abstract])) OR (UBE3A-AS[Title/Abstract])) OR (UBE3A-AS1[Title/Abstract])) OR (UBE3A-ATS[Title/Abstract])) OR (UBE3AATS[Title/Abstract]))

**#5 #3 AND #4**

**Cochrane Library**

**#1** MeSH descriptor: [Neoplasms] explode all trees

**#2** **(**Tumor OR Neoplasm OR Tumors OR Neoplasia OR Neoplasias OR Cancer OR Cancers OR Malignant Neoplasm OR Malignancy OR Malignancies OR Malignant Neoplasms OR (Neoplasm, Malignant) OR (Neoplasms, Malignant) OR (Benign Neoplasms) OR (Benign Neoplasm) OR (Neoplasms, Benign) OR (Neoplasm, Benign): ti,ab,kw

**#3 #1 OR #2**

**#4** (SNHG14 OR small nucleolar RNA host gene 14 OR 115HG OR IC-SNURF- SNRPN OR LNCAT OR NCRNA00214 OR U-UBE3A-ATS OR UBE3A-AS OR UBE3A-AS1 OR UBE3A-ATS OR UBE3AATS):ti,ab,kw

**#5 #3 AND #4**

**Embase**

**#1** ' Neoplasms '/exp

**#2** 'Tumor':ab,ti OR 'Neoplasm':ab,ti OR ' Tumors ':ab,ti OR ' Neoplasia ':ab,ti OR ' Neoplasias ':ab,ti OR ‘Cancer’:ab,ti OR ' Cancers ':ab,ti OR ' Malignant Neoplasm ':ab,ti OR ' Malignancy ':ab,ti OR ' Malignancies ':ab,ti OR ' Malignancies ':ab,ti OR ' Malignant Neoplasms ':ab,ti OR ' Neoplasm, Malignant ':ab,ti OR ‘Neoplasms, Malignant’:ab,ti OR ' Benign Neoplasms ':ab,ti OR ' Benign Neoplasm ':ab,ti OR ' Neoplasms, Benign ':ab,ti

**#3 #1 OR #2**

**#4** ' SNHG14': ab,ti OR ' small nucleolar RNA host gene 14':ab,ti OR ' 115HG ':ab,ti OR ' IC-SNURF- SNRPN ':ab,ti OR ' LNCAT ':ab,ti OR ‘NCRNA00214’:ab,ti OR ' U-UBE3A-ATS ':ab,ti OR ' UBE3A-AS ':ab,ti OR ' UBE3A-AS1 ':ab,ti OR ' UBE3A-ATS ':ab,ti OR ' UBE3AATS ':ab,ti

**#5 #3 AND #4**

**Web of science**

**#1** Neoplasms[Topic] OR Tumor[Topic]) OR (Neoplasm[Topic])) OR (Tumors[Topic])) OR (Neoplasia[Topic])) OR (Neoplasias[Topic])) OR (Cancer[Topic])) OR (Cancers [Topic])) OR (Malignant Neoplasm [Topic])) OR (Malignancy [Topic])) OR (Malignancies[Topic])) OR (Malignant Neoplasms[Topic])) OR (Neoplasm, Malignant [Topic])) OR (Neoplasms, Malignant[Topic])) OR (Benign Neoplasms[Topic])) OR (Benign Neoplasm[Topic])) OR (Neoplasms, Benign[Topic])) OR (Neoplasm, Benign[Topic])

**#2** SNHG14[Topic]) OR (small nucleolar RNA host gene 14[Topic])) OR (115HG[Topic])) OR (IC-SNURF- SNRPN[Topic])) OR (LNCAT[Topic])) OR (NCRNA00214[Topic])) OR (U-UBE3A-ATS[Topic])) OR (UBE3A-AS[Topic])) OR (UBE3A-AS1[Topic])) OR (UBE3A-ATS[Topic])) OR (UBE3AATS[Topic]))

**#3 #1 AND #2**
